# Supplementary material for: Evaluation of phosphate rock as the only source of phosphorus for the growth of tall and semi-dwarf durum wheat and rye plants using digital phenotyping
Source: PeerJ. 2023 Aug 29;11:e15972. doi: 10.7717/peerj.15972 (PMC10473039; doi:10.7717/peerj.15972)
Supplement: Supplemental Information 1 [file peerj-11-15972-s001.docx]

**Table S1.** Protocol of Hoagland solution (HS) and HS without phosphate

| **Hoagland solution**  **(for normal nutrition control)** | | | |
| --- | --- | --- | --- |
|  | **Concentration** | | **Mixed stock solution #*** |
|  | **mM** | **mg/L** |  |
| KNO_3_ | 5 | 505 | 1 |
| Ca(NO_3_)_2_ x 4 H_2_O | 5 | 1180 | 1 |
| MgSO_4_ x 7 H_2_O | 2 | 493 | 2 |
| MnCl_2_ x 4 H_2_O | 0.0091 | 1.81 | 2 |
| ZnSO_4_ x 7 H_2_O | 0.0008 | 0.22 | 2 |
| CuSO_4_ x 5 H_2_O | 0.0003 | 0.08 | 2 |
| H_3_BO_3_ | 0.0463 | 2.86 | 3 |
| Ferric sodium EDTA (CAS Number: 15708-41-5) | 0.014 | 5 | 3 |
| Na_2_MoO_4_ x 2 H_2_O | 0.0005 | 0.12 | 3 |
| K-PO_4_ | 1 | 117.3 mg KH_2_PO_4_ + 31.5 mg K_2_HPO_4_ x 2 H_2_O ** | 4 |
|  |  |  |  |
| **Hoagland solution without phosphate**  **(for pots with phosphorite powder and for no-phosphorous control pots)** | | | |
|  | **Concentration** | | **Mixed stock solution #** |
|  | **mM** | **mg/L** |  |
| KNO_3_ | 5 | 505 | 1 |
| Ca(NO_3_)_2_ x 4 H_2_O | 5 | 1180 | 1 |
| MgSO_4_ x 7 H_2_O | 2 | 493 | 2 |
| MnCl_2_ x 4 H_2_O | 0.0091 | 1.81 | 2 |
| ZnSO_4_ x 7 H_2_O | 0.0008 | 0.22 | 2 |
| CuSO_4_ x 5 H_2_O | 0.0003 | 0.08 | 2 |
| H_3_BO_3_ | 0.0463 | 2.86 | 3 |
| Ferric sodium EDTA (CAS Number: 15708-41-5) | 0.014 | 5 | 3 |
| Na_2_MoO_4_ x 2 H_2_O | 0.0005 | 0.12 | 3 |

* - For the ingredients labeled by the same # of mixed stock solution the combined stock solutions (1000 time more concentrated than listed in table) were prepared. These solutions were added 1 ml each to 997 ml of distilled water just before use.

** - Combination of mono- and dipotassium phosphate was used to obtain an optimal pH of the solution
