# Supplementary figures and images for: Evaluation of phosphate rock as the only source of phosphorus for the growth of tall and semi-dwarf durum wheat and rye plants using digital phenotyping
Source: PeerJ. 2023 Aug 29;11:e15972. doi: 10.7717/peerj.15972 (PMC10473039; doi:10.7717/peerj.15972)

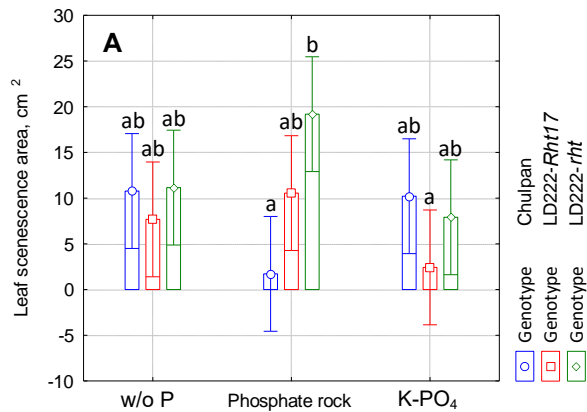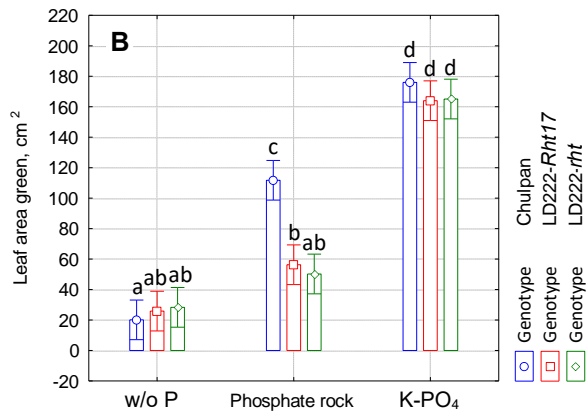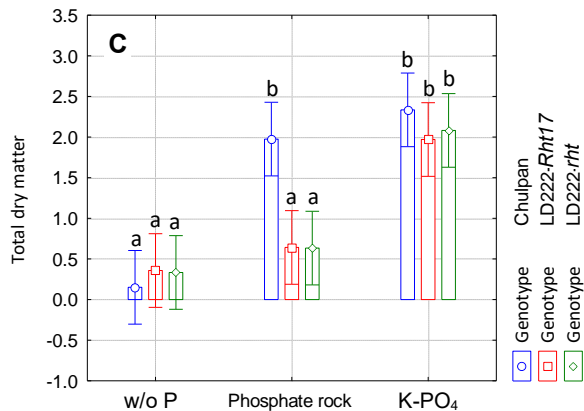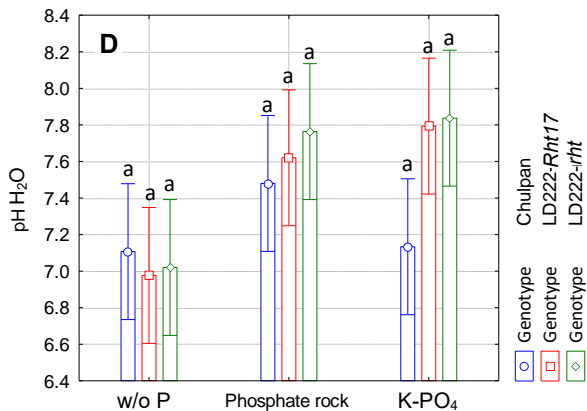

Supplement: Supplemental Information 2 — (A) Leaf senescence area. (B) Green leaf area. (C) Total dry matter of plants. (D) The pH of substrate water extract. The points designate the marginal means, the letters above the points designate the homogenous groups according to Tukey HSD test at α=0.05. [file peerj-11-15972-s002.pdf]

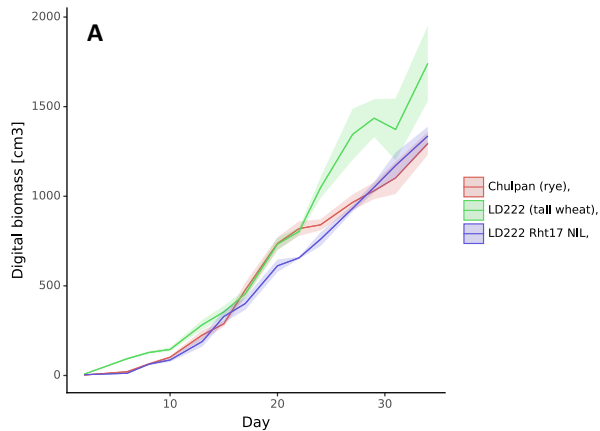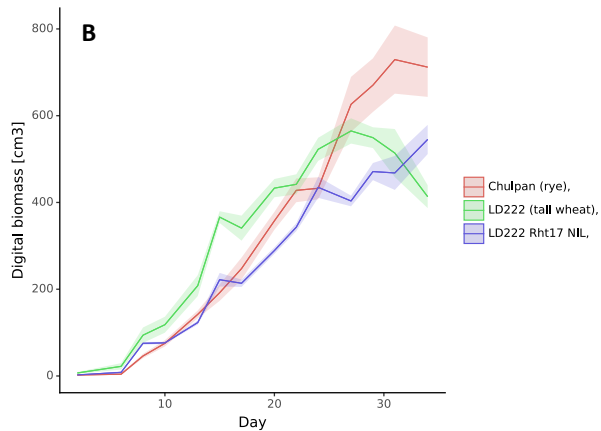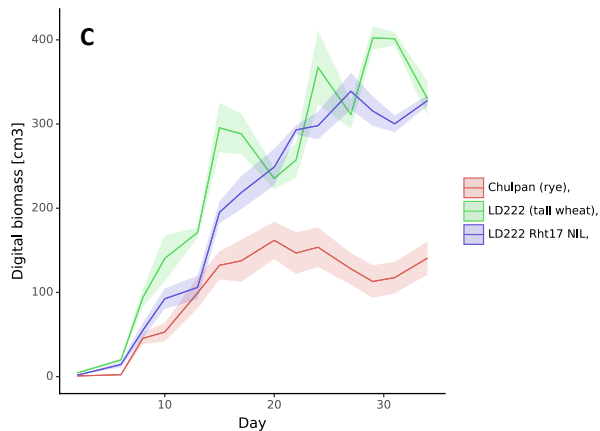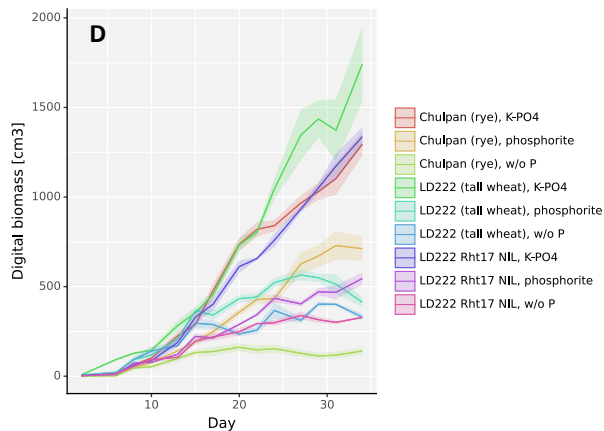

Supplement: Supplemental Information 3 — The means are represented by solid color lines. The standard errors are shown as faint-color ribbons above and below the lines. Non-overlapping one standard error intervals may be interpreted as significant difference of means at α=0.05. The x axis indicates the day after emergence. Nutrition variants: (A) soluble phosphates, (B) phosphate rock powder, (C) without phosphates, (D) all variants together. [file peerj-11-15972-s003.pdf]

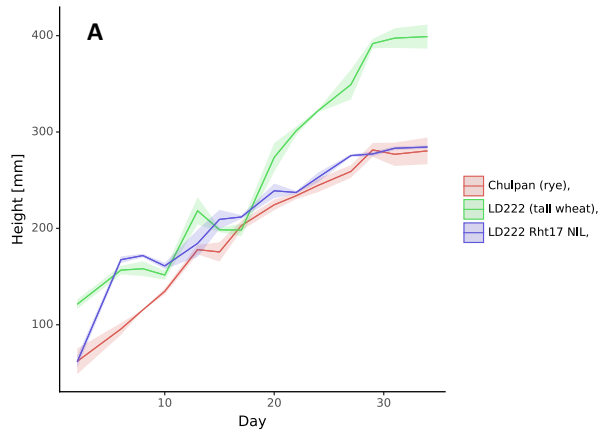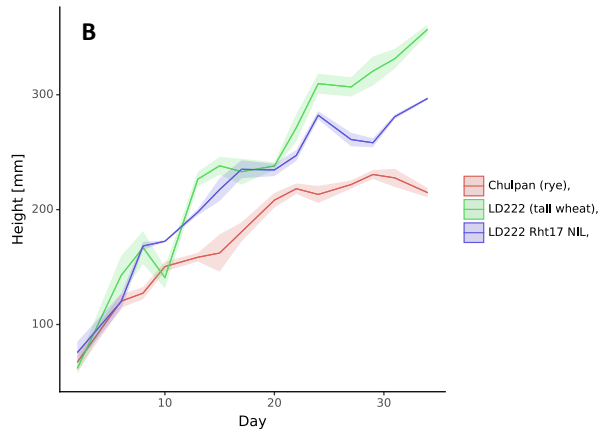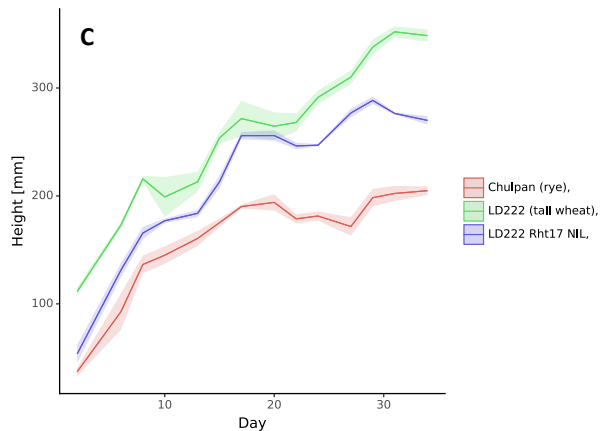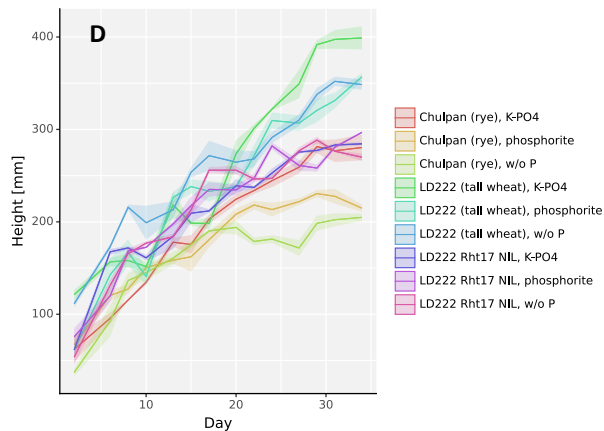

Supplement: Supplemental Information 4 — The means are represented by solid color lines. The standard errors are shown as faint-color ribbons above and below the lines. Non-overlapping one standard error intervals may be interpreted as significant difference of means at α=0.05. The x axis indicates the day after emergence. Nutrition variants: (A) soluble phosphates, (B) phosphate rock powder, (C) without phosphates, (D) all variants together. [file peerj-11-15972-s004.pdf]

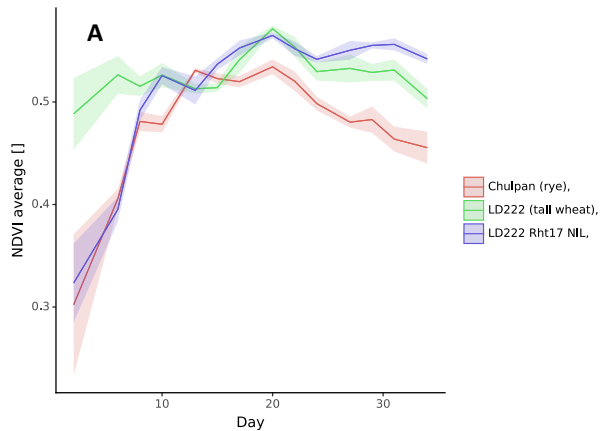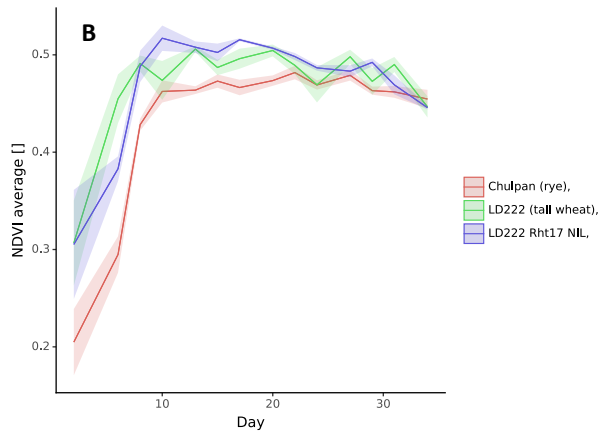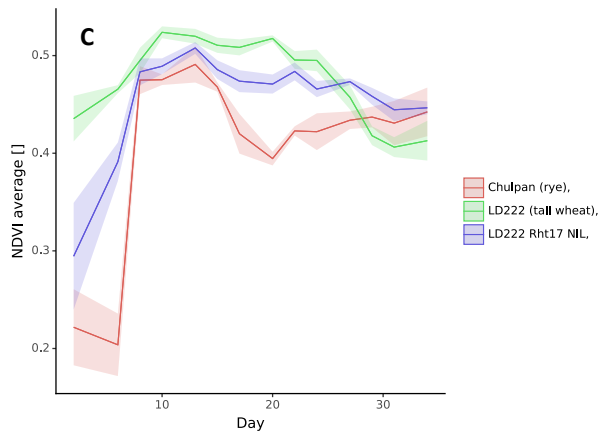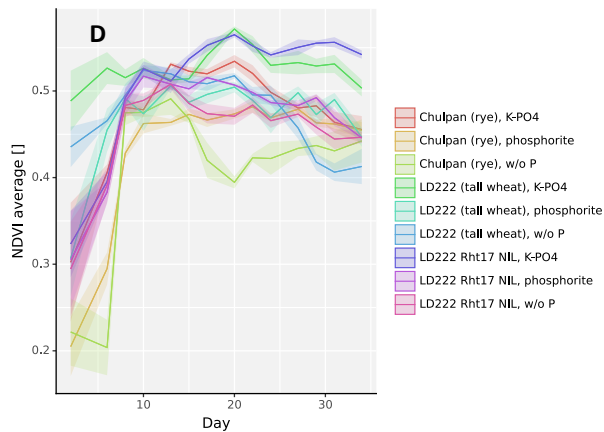

Supplement: Supplemental Information 5 — The means are represented by solid color lines. The standard errors are shown as faint-color ribbons above and below the lines. Non-overlapping one standard error intervals may be interpreted as significant difference of means at α=0.05. The x axis indicates the day after emergence. Nutrition variants: (A) soluble phosphates, (B) phosphate rock powder, (C) without phosphates, (D) all variants together. [file peerj-11-15972-s005.pdf]

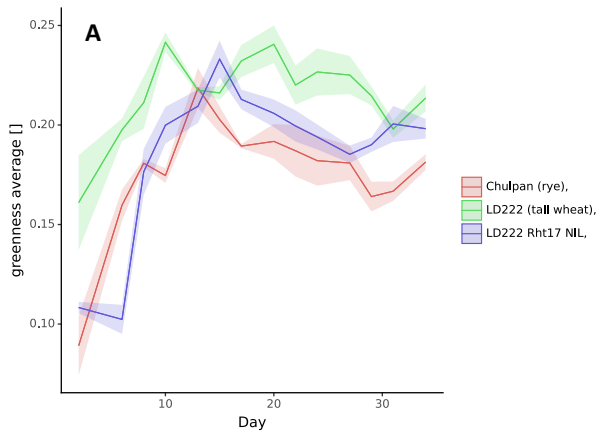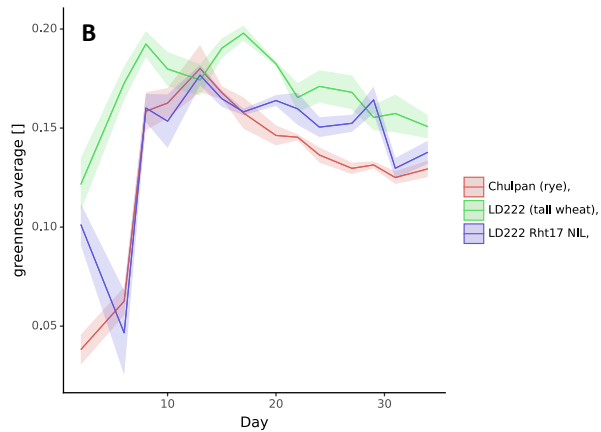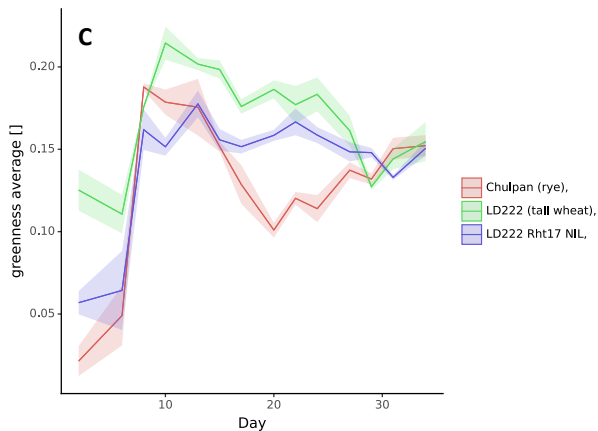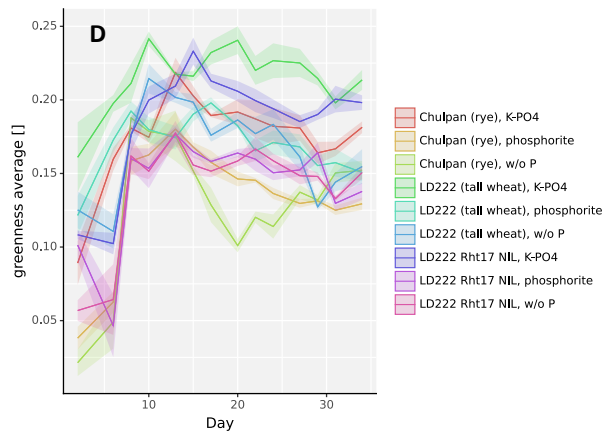

Supplement: Supplemental Information 6 — The means are represented by solid color lines. The standard errors are shown as faint-color ribbons above and below the lines. Non-overlapping one standard error intervals may be interpreted as significant difference of means at α=0.05. The x axis indicates the day after emergence. Nutrition variants: (A) soluble phosphates, (B) phosphate rock powder, (C) without phosphates, (D) all variants together. [file peerj-11-15972-s006.pdf]

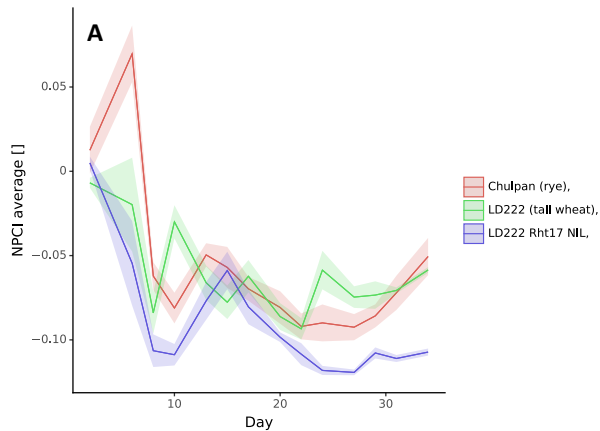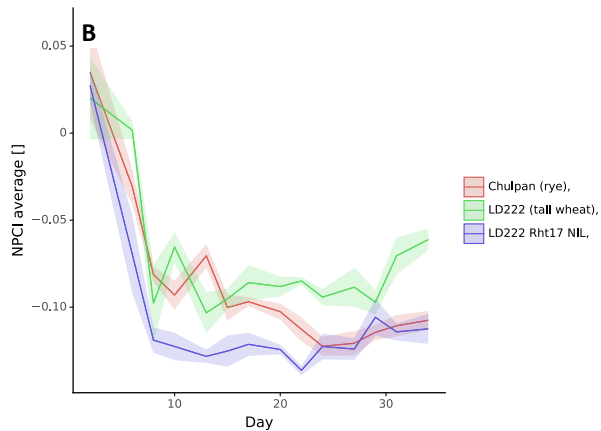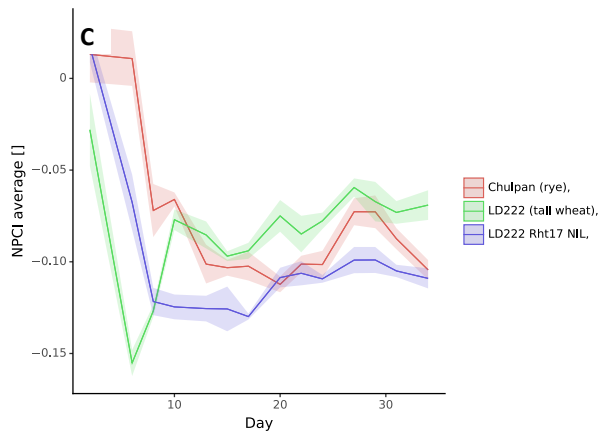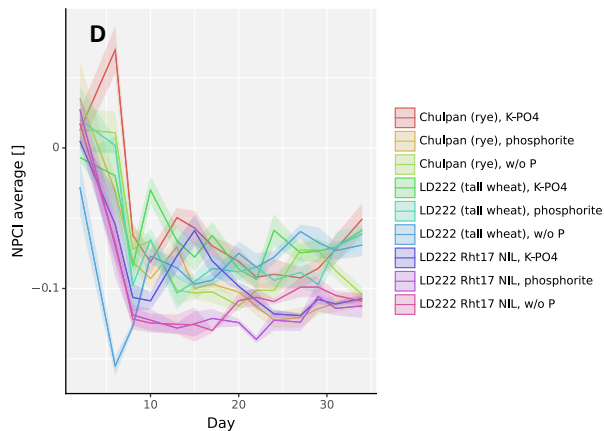

Supplement: Supplemental Information 7 — The means are represented by solid color lines. The standard errors are shown as faint-color ribbons above and below the lines. Non-overlapping one standard error intervals may be interpreted as significant difference of means at α=0.05. The x axis indicates the day after emergence. Nutrition variants: (A) soluble phosphates, (B) phosphate rock powder, (C) without phosphates, (D) all variants together. [file peerj-11-15972-s007.pdf]

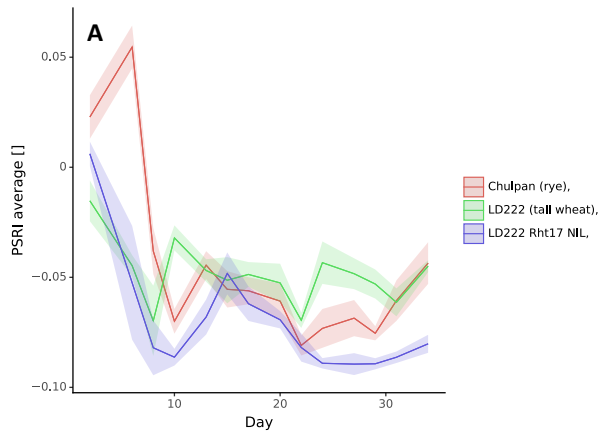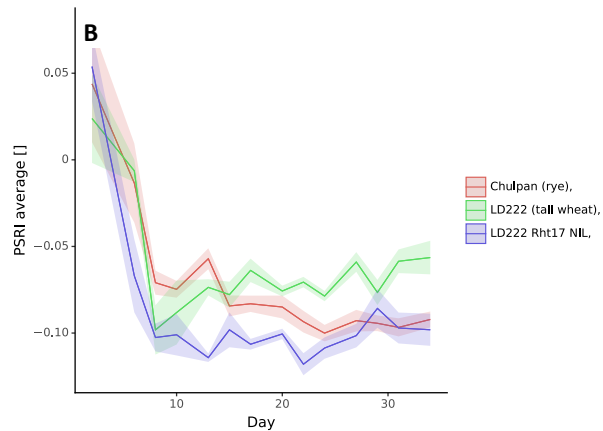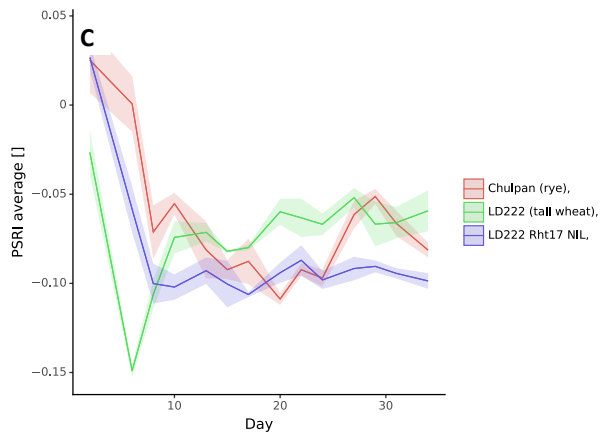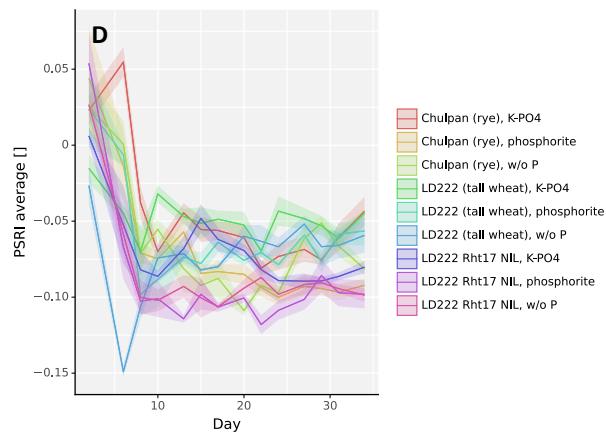

Supplement: Supplemental Information 8 — The means are represented by solid color lines. The standard errors are shown as faint-color ribbons above and below the lines. Non-overlapping one standard error intervals may be interpreted as significant difference of means at α=0.05. The x axis indicates the day after emergence. Nutrition variants: (A) soluble phosphates, (B) phosphate rock powder, (C) without phosphates, (D) all variants together. [file peerj-11-15972-s008.pdf]
